# Complexation-Based Detection of Nickel(II) at a Graphene-Chelate Probe in the Presence of Cobalt and Zinc by Adsorptive Stripping Voltammetry

**DOI:** 10.3390/s17081711

**Published:** 2017-07-25

**Authors:** Keagan Pokpas, Nazeem Jahed, Priscilla G. Baker, Emmanuel I. Iwuoha

**Affiliations:** SensorLab, Department of Chemistry, University of the Western Cape, Bellville 7535, South Africa; 2874933@myuwc.ac.za (K.P.); pbaker@uwc.ac.za (P.G.B.); eiwuoha@uwc.ac.za (E.I.I.)

**Keywords:** graphene, trace metal analysis, dimethylglyoxime, adsorptive stripping voltammetry, nickel determination, Nafion

## Abstract

The adsorptive stripping voltammetric detection of nickel and cobalt in water samples at metal film electrodes has been extensively studied. In this work, a novel, environmentally friendly, metal-free electrochemical probe was constructed for the ultra-trace determination of Ni^2+^ in water samples by Adsorptive Cathodic Stripping Voltammetry (AdCSV). The electrochemical platform is based on the adsorptive accumulation of Ni^2+^ ions directly onto a glassy carbon electrode (GCE) modified with dimethylglyoxime (DMG) as chelating agent and a Nafion-graphene (NGr) nanocomposite to enhance electrode sensitivity. The nafion-graphene dimethylglyoxime modified glassy carbon electrode (NGr-DMG-GCE) shows superior detection capabilities as a result of the improved surface-area-to-volume ratio and enhanced electron transfer kinetics following the incorporation of single layer graphene, while limiting the toxic effects of the sensor by removal of the more common mercury, bismuth and lead films. Furthermore, for the first time the NGr-DMG-GCE, in the presence of common interfering metal ions of Co^2+^ and Zn^2+^ demonstrates good selectivity and preferential binding towards the detection of Ni^2+^ in water samples. Structural and morphological characterisation of the synthesised single layer graphene sheets was conducted by Raman spectrometry, HRTEM and HRSEM analysis. The instrumental parameters associated with the electrochemical response, including accumulation potential and accumulation time were investigated and optimised in addition to the influence of DMG and graphene concentrations. The NGr-DMG-GCE demonstrated well resolved, reproducible peaks, with RSD (%) below 5% and a detection limit of 1.5 µg L^−1^ for Ni^2+^ reduction at an accumulation time of 120 s. The prepared electrochemical sensor exhibited good detection and quantitation towards Ni^2+^ detection in tap water samples, well below 0.1 mg L^−1^ set by the WHO and EPA standards. This is comparable to the South African drinking water guidelines of 0.15 mg L^−1^.

## 1. Introduction

The considerable increase in technological advancement has led to a growing demand for accurate, analytical monitoring of trace levels of metal ions in drinking and wastewater samples. While nickel, cobalt and zinc, are three common trace elements essential for human health, they may also exhibit toxic effects in humans as a result of their non-biodegradable nature and long biological half-life [1,2]. At high concentrations they have been linked to a variety of illnesses including cardiovascular diseases, cancer and skin allergies such as dermatitis. Ni, Co and Zn are commonly found in water samples due to their use in alloys and in electroplating of materials to enhance mechanical properties and corrosion resistance. Dangerous exposures and illnesses may be minimized by limiting common sources of exposure and developing accurate early detection methods [3]. The maximum allowable limits for Ni^2+^ detection in tap water samples has been set at 0.1 mg L^−1^ by the world health organization (WHO) [4] and the United States environmental protection agency (US-EPA) and at 0.15 mg L^−1^ in the South African drinking water guidelines [5].

The use of stripping analysis has significantly increased over the last two decades and has proved to be a highly sensitive method for the ultra-trace determination of metal ions. The simplicity and rapidness of the electrochemical analytical techniques, ability for in-situ pre-concentration steps [6,7] and low cost and reliable nature [8] makes it an attractive alternative to the more common chromatographic and atomic absorption spectrometric methods. Anodic stripping voltammetry (ASV), cathodic stripping voltammetry (CSV) and adsorptive stripping voltammetry (AdSV) are three common examples of conventional stripping methods. Here, electrolytic deposition and stripping at metal-based electrodes are among the most sensitive electrochemical sensing techniques. AdSV, suitable for samples in which preconcentration cannot be controlled by electrolysis [9] has been shown to be a highly sensitive technique for the simultaneous and individual detection of Ni and Co. Commonly, the technique is based on the accumulation of analyte species, in the presence of a suitable complexing agent, on an electroplated metal film. In contrast to most metal ions, the detection of Ni is often complicated and associated with low electrochemical signals [10]. Complexation with organic ligands has shown to significantly enhance the sensitivity and selectivity of electrochemical sensors for Ni^2+^ determination. A wide range of complexing agents have been employed in stripping analysis and are highly dependent on the application. Dioximes such as, nioxime [11,12,13] and more popularly dimethylglyoxime (DMG) [8,10,14,15,16,17,18,19,20,21,22,23,24] have gained widespread attention.

Mercury film (MF) [25,26], lead film (LF) [11,15,27] and bismuth film (BF) [28,29,30,31] electrodes, prepared by ex-situ and in-situ plating techniques have been widely employed as alternatives to the highly toxic hanging mercury drop electrodes (HMDE) and their development for analytical applications is still of great interest. While more environmentally friendly and of lower toxic nature, the MFE, LFE and BFE still pose toxic effects in the environment, owing to their non-biodegradable nature. As a result, manufacturing of electrochemical platforms without the use of metal films may result in green, non-toxic sensing techniques for future applications.

The use of chemically modified electrodes (CME) has garnered tremendous interest amongst analytical chemists in recent times and is one such method that holds great potential for alleviating the use of metallic films. CMEs have chemically selective functional groups attached to the electrode surface for improved selectivity and sensitivity [8]. The CMEs have particularly demonstrated their usefulness in analytical systems where detection at mercury or metal based electrodes by conventional stripping analysis is not possible. For example, in cases where the analyte is sparingly soluble in mercury or lacks amalgam formation with metal ions. A major advantage of CMEs is their ability to incorporate a wide range of chemical modifiers onto the substrate with minimum effort. The application of CMEs for the detection of metal ions, in conjunction with metal films at the drop coated glassy carbon (GC) [19,22,23,24,32] and modified carbon paste electrodes (CPE) [18,20] have been demonstrated. CMEs show improved electrode selectivity and higher resistance to surface active compounds and intermetallic interferences [24]. Zen et al., for the first time reported the use of a novel Nafion-coated mercury film electrode (NCMFE) by, incorporating chelating agents directly onto the NCMFE to improve analyte sensitivity and selectivity for the detection of trace metals by SWASV [22]. Their results showed that binding chelating agents directly onto the electrode substrate limits fouling from organic interferents when compared to in-situ analysis. Current, research has expanded to include mercury or metal-free sensors relying on CMEs. Bing et al., reported the use of a glassy carbon electrode modified with polymers containing DMG as a mercury-free sensor for detection of Ni^2+^ ions [8]. In the research conducted by Gonzalez et al., and Tartarotti et al., carbon paste electrodes modified with chelating agents were employed as alternative modified electrodes for the detection of Ni^2+^ in water and fuel ethanol samples respectively. Their research exhibited for the first time, the applicability of modified electrochemical sensors for the detection of metal ions by AdSV in the absence of metallic films [18,20]. Their results showed detection limits comparable to the common metal-based transducers at shorter analysis times. Ferancova et al., further proposed the use of screen printed electrodes modified with Nafion and DMG as a low cost, robust metal-free sensor for the detection of Ni^2+^ in industrial discharge water [16]. Although metal-free CMEs have shown good selectivity and long-term stability, a major drawback is that longer accumulation times are often required to achieve sensitivities comparable to their metallic counterparts.

Graphene, a 2-D allotrope of carbon has found widespread application in electrochemical sensor technology owing to its ability to enhance electrode sensitivity. Graphene’s large surface-area-to-volume ratio, enhanced electrocatalytic effects and enhanced electron transfer kinetics makes it suitable for electrochemical sensors for various applications [33,34]. Extensive research has been conducted in the use of graphene-based sensors for detection of trace metal ions by stripping voltammetric techniques [33,35,36,37,38,39,40,41]. To date however, no work has been reported on the use of graphene for determination of trace Ni^2+^ at an in-situ or chemically modified DMG electrode by AdSV.

A Nafion semi-permeable membrane, has been employed as a binder for electrode coatings in a variety of sensor applications. However, it assists in reducing the effects of adsorbed surfactants at the electrode surface, provides mechanical stability to coated films and utilizes its cation exchanger ability to exclude anion interferences [16,22,32,35].

In this work we report, for the first time, a highly selective, voltammetric chemically-modified electrode for the determination of Ni^2+^ in water samples, in the presence of Co^2+^ and Zn^2+^. The sensor is based on a glassy carbon electrode modified with dimethylglyoxime as chelating agent to enhance analyte selectivity and a Nafion-graphene nanocomposite to enhance electrode sensitivity, in the absence of an electroplated metal film. To date no work has been reported on the use of a Nafion-graphene-dimethylglyoxime nanocomposite CME for the detection of Ni^2+^ in water by AdCSV.

## 2. Materials and Methods

### 2.1. Chemicals and Reagents

All chemicals used in the study were of analytical reagent grade. Ultra-pure distilled water (Millipore, Billerica, MA, USA) was used to prepare all solutions. Nafion perfluorinated resin solution 5 wt % in lower aliphatic alcohols and water and 2,3-butanedione dioxime (dimethylglyoxime) were purchased from Aldrich (St. Louis, MO, USA). Ni^2+^ standard stock solutions (1000 mg L^−1^, atomic absorption standard solution) and all other metal standards were obtained from Sigma-Aldrich and diluted as required. Natural graphite powder (microcrystal grade, 99.9995%, Metal base) UCP1-M grade, Ultra “F” purity was purchased from Alfa-Aesar (Haverhill, MA, USA).

Ammonia/ammonium chloride (NH_3_/NH_4_Cl) buffer solution (0.1 M, pH 9.3) was used as supporting electrolyte and prepared by mixing appropriate quantities of ammonia (NH_3_) and ammonium chloride (NH_4_Cl). An 827 pH lab pH meter (Metrohm, Cape Town, South Africa) was calibrated using pH 4 and 7 calibration buffer solutions and then used to verify the pH of the prepared NH_3_/NH_4_Cl buffer solution. Ferricyanide solutions were prepared by dissolving potassium ferricyanide (K_3_Fe(CN)_6_) salt in 1 M KCl solution.

### 2.2. Apparatus

All electrochemical voltammetric experiments were performed with a Metrohm Autolab PGSTAT101 instrument, in combination with the Nova 1.11 Software and controlled by a personal computer. A conventional three-electrode electrochemical system, consisting of a nafion graphene modified dimethylglyoxime glassy carbon electrode (NGr-DMG-GCE) serving as working electrode was utilized for all electrochemical studies, unless stated otherwise. An Ag/AgCl (saturated with KCl) and platinum wire served as reference and counter electrodes, respectively. All experiments were performed in a one compartment 20 mL voltammetric cell at room temperature.

Fourier Transform Infrared (FT-IR) spectra were recorded using a Spectrum 100 spectrometer (Perkin Elmer, Waltahm, MA, USA) coupled to an Attenuated Total Reflectance (ATR) sample holder. FT-IR was used to confirm the presence of oxime groups in the dimethylglyoxime structure. Appropriate quantities of ground KBr and samples (DMG) were mixed and pressed in a handheld hydraulic press to prepare KBr pellets. High Resolution Scanning Electron Microscopy (HRSEM) measurements were performed using an Auriga HRSEM instrument equipped with Electronic Data System (EDS) (Woburn, MA, USA). The samples were dried in a vacuum oven and deposited on the silicon grid surface before HRSEM observations. High Resolution Transmission Electron Microscopy (HRTEM) measurements were carried out with a Tecnai G2 F20X-Twin MAT Field Emission Transmission Electron Microscope from FEI (Eindhoven, The Netherlands) under an acceleration voltage of 200 kV. The samples were prepared by dropping a dilute suspension of graphene in ethanol onto copper grids followed by air drying at room temperature. Raman spectroscopy was obtained using a LabRam HR by Jobin-Yvon Horiba scientific (Explora, France) in conjunction with a Model BX41 microscope (Olympus, Irvine, CA, USA) at 532 nm wavelength. Samples were prepared by drop coating graphene suspensions or thin graphene films onto glass slides and allowing to dry at room temperature. A Zahner-Elektrik Electrochemical Workstation IM6e from Zahner-Elektrik GmbH & CoKG (Kronach, Germany) was used for electrochemical impedance spectroscopy (EIS) measurements in 5 mM K_3_Fe(CN)_6_ in 1 M KCl as electrolyte solution.

### 2.3. Preparation of the Nafion-Graphene Dimethylglyoxime Suspension (NGr-DMG)

All graphene samples used in this work were prepared by chemical reduction of graphene oxide with sodium borohydride (NaBH_4_) as reducing agent. Appropriate quantities of graphene oxide, prepared using a modified Hummer method [42], sodium borohydride and distilled water were mixed for 30 min followed by heating to 135 °C under reflux for 3 h. The resulting black precipitate was centrifuged, washed with water, ethanol and dried in a vacuum oven as described in the work by Shen et al. [35,43]. NGr-DMG suspensions were then prepared by mixing appropriate quantities of Nafion (5 wt %), prepared graphene and dimethylglyoxime powder in an ethanol solvent followed by ultrasonication for 1 h.

### 2.4. Preparation of Nafion-Graphene Dimethylglyoxime Modified Glassy Carbon Electrode (NGr-DMG-GCE)

Glassy carbon electrodes (Bioanalytical Systems inc., BASi, West Lafayette, IN, USA) with area of 0.071 cm^2^ (3 mm diameter) was polished with slurries of alumina powder (1, 0.3 and 0.05 µm). The electrode was rinsed with ultra-pure distilled water, successively sonicated for 5 min in Ethanol and ultra-pure water respectively and dried at room temperature. The polished surface of the GCE was then coated with 4 µL of the prepared NGr-DMG suspension and allowed to dry at room temperature in order to allow the solvent to evaporate to form the NGr-DMG-GCE. No further electrode pre-treatment was required.

### 2.5. Procedure for Square Wave Adsorptive Cathodic Stripping Voltammetric (AdCSV) Analysis

The three-electrode system with NGr-DMG-GCE as working electrode was immersed into an electrochemical cell containing 20 mL NH_3_/NH_4_Cl buffer solution (0.1 M, pH 9.3), degassed with N_2_ gas for 5 min, as supporting electrolyte. Appropriate volumes of 10 ppm Ni^2+^ stock solution was added prior to analysis. A reduction potential of −0.7 V was applied to the working electrode, under constant stirring for 120 min to aid with accumulation of the metal ions at the electrode surface. After a brief rest period, the potential of the NGr-DMG-GCE was cathodically scanned between −0.7 and −1.3 V by applying a square-wave waveform. No further electrochemical cleaning was required in between runs. NH_3_/NH_4_Cl buffer solution was used as test solutions for all experiments.

### 2.6. Sample Preparation

All tap water samples were collected from our laboratory at the University of the Western Cape (Bellville, South Africa) after allowing the tap to run for 1 min. The tap water samples used for nickel detection were diluted for characterization by SWAdCSV by mixing a 9 mL sample of tap water and 1 mL of 1 M NH_3_/NH_4_Cl buffer. The prepared tap water samples were added to the electrochemical cell and the analysis was performed as described by the procedure in Section 2.5.

## 3. Results and Discussion

### 3.1. Fourier Transform Infrared Spectroscopy (FT-IR) of DMG

**Figure 1:**
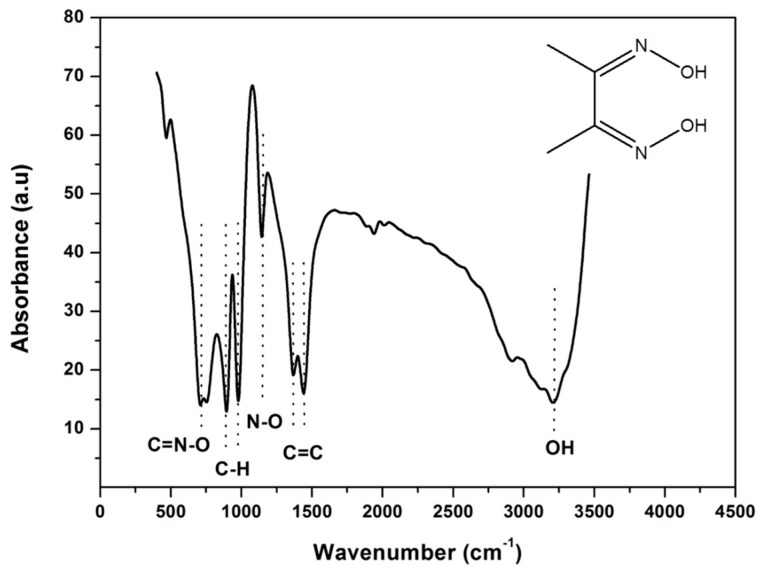
Fourier transform infrared spectrometry of dimethylglyoxime as a KBr pellet. Inset: schematic representation of the dimethylglyoxime structure.

The presence of functional groups in the chelating agent (dimethylglyoxime, DMG) was analyzed using FT-IR analysis. The spectrum of DMG was measured between 4000 and 450 cm^−1^ and presented in Figure 1. The infrared spectrum exhibited distinct absorption bands at 3205, 1441, 1144, 979 and 716 cm^−1^ which are attributed to ⱱ (OH), ⱱ (C=C), ⱱ (N-O), ⱱ (C-H) aliphatic and ⱱ (C=N-O) stretching frequencies respectively. The FT-IR spectrum confirms the presence of the oxime groups (NOH) in the dimethylglyoxime molecule which facilitates the complex formation with Ni^2+^ ions. Similar results have been reported in the work conducted by Shaker et al. [44,45,46].

### 3.2. Nickel Dimethylglyoxime (Ni(DMG)_2_) Complex Formation and Electrochemical Stripping Reduction

**Figure 2:**
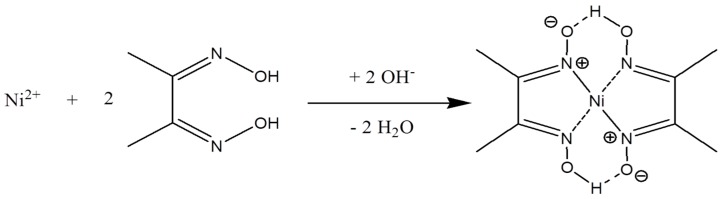
Schematic illustration of the metal-chelate complex formation [46].

The 1:2 complex formation of Ni^2+^ with dimethylglyoxime, C_4_H_6_(NOH)_2_ has been extensively studied in the gravimetric and electrochemical determination of nickel [47]. Figure 2 shows a schematic representation of the above mentioned chelation reaction. Here, electron pairs are donated to the Ni^2+^ ions by nitrogen atoms of the dimethylglyoxime molecule and not the oxygen atoms as commonly expected. In addition, one proton is lost from a oxime group on each of the dimethylglyoxime molecules [48]. The addition of OH^−^ anions facilitates metal-chelate complex formation [49] which occurs quantitatively in solutions buffered between pH’s of 5 and 9. Ammonia or citrate buffer is required to facilitate the reaction by preventing a pH drop below 5. At pH values lower than 5 a disturbance in the equilibria of the reaction occurs, favoring the formation of Ni^2+^ and causing the dissolution of the [Ni(DMGH)_2_] complex. Previous research has shown that the [Ni(DMGH)_2_] complex has a square planar geometry [47,50] with nitrogen atoms situated at the corners of a square in a single plane, positioned around a central nickel atom [50].

The exact nature of the adsorption and reduction of Ni^2+^ in the AdSV technique has been widely debated and remains a highly controversial topic. Traditionally complexation of Ni^2+^ with DMG occurs in solution, prior to its subsequent adsorption onto an electrochemically plated metal film. In the NGr-DMG-GCE sensor, bound DMG confines [Ni(DMGH)_2_] complex formation and pre-concentration directly at the surface of the electrode (not in the solution), simplifying the adsorption process (Equation (1)) [51,52].

(1) $${\mathrm{Ni}}^{2+}+2{\mathrm{DMGH}}_{2}+2{\mathrm{OH}}^{-}\to \left[\mathrm{Ni}{\left(\mathrm{DMGH}\right)}_{2}\right]+2H_{2}O\;(\mathrm{Pre-concentration}\;\mathrm{step})$$

(2) $$\left[\mathrm{Ni}{\left(\mathrm{DMGH}\right)}_{2}\right]+10e^{-}+10H^{+}\to {\mathrm{Ni}}^{2+}+2\mathrm{DHAB}\;(\mathrm{Stripping}/\mathrm{reduction}\;\mathrm{step})$$

The subsequent electrochemical reduction of the [Ni(DMGH)_2_] complex is shown in Equation (2). It involves the overall reduction of both the central metal atom and the surrounding ligands in an overall 10-electron reduction process giving rise to 2,3-bishydroxylaminebutane, a stable electrolysis product [51,52]. The reacted [Ni(DMGH)_2_] complex is reduced and subsequently moves away from the electrode surface, while unconverted DMG remains present for further complexation with metal cations.

### 3.3. Characterization of Graphene (Gr)

**Figure 3:**
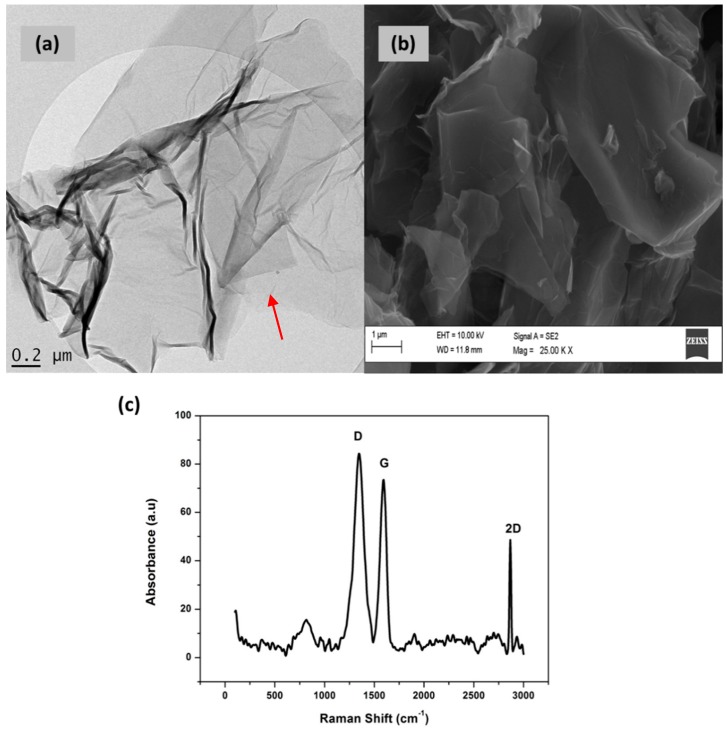
Selected structural and morphological characterization of synthesized graphene: (**a**) high resolution transmission electron microscopy (HRTEM); (**b**) high resolution scanning electron microscopy (HRSEM) and (**c**) raman spectroscopy.

The structural and morphological characterization of the synthesized graphene was studied using HRTEM, HRSEM and Raman spectroscopy and is shown in Figure 3. Figure 3a shows the HRTEM image of the synthesized graphene after ultrasonication in ethanol and drop coated onto holey mesh Cu grids. The HRTEM image, at low magnification, shows large transparent single layer sheets with a few wrinkles for the chemically synthesized graphene. Single layer graphene can be observed by a lack in stacking of successive sheets, as shown by the arrow in Figure 3a. The HRSEM image of graphene in Figure 3b shows aggregated flakes, randomly arranged and packed on top of one another. Both HRSEM and HRTEM images confirm the presence of good quality single layer graphene. Raman spectroscopy, one of the most important tools for the characterization of graphene, was used to confirm the synthesis of single layer graphene sheets. The Raman spectrum, Figure 3c, reveals three distinct peaks at 1349 cm^−1^, 1591 cm^−1^ and 2863 cm^−1^ which are attributed to the D, G and 2D bands respectively. The G-band, found at 1591 cm^−1^, is common in all sp^2^ carbon systems and arises from the in plane stretching of the C-C bond. The D-mode (1349 cm^−1^) occurs in the resonance Raman spectra as a result of the disorder in the sp^2^ hybridized carbon system of the graphene structure. An I_D_/I_G_ ratio of 1.21 confirms the removal of oxygen from the graphene structure. A strong 2D band is observed in the range of 2500–2900 cm^−1^ for sp^2^ hybridized carbon materials. This 2D-band can be found at double the frequency of the D-band and occurs as a result of the second order two phonon Raman scattering processes. The intense and sharp 2D-band at 2863 cm^−1^ confirms the presence of single layer graphene sheets over multi-layer graphene structures.

### 3.4. Morphological Characterization of Modified Electrode

**Figure 4:**
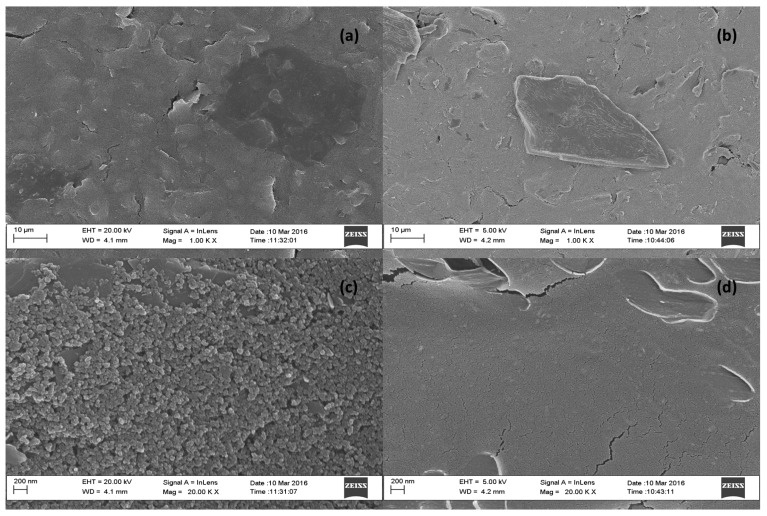
High resolution scanning electron microscope (HRSEM) image of (**a**,**c**) Bare SPCE and (**b**,**d**) NGr-DMG-SPCE at 1000× and 20,000× magnification.

**Figure 5:**
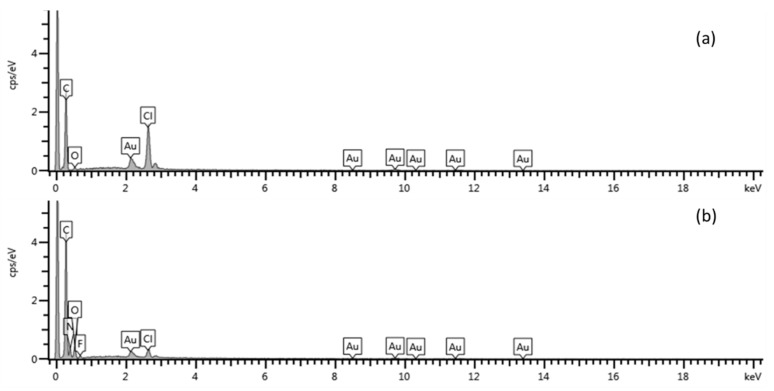
Energy dispersive spectroscopy (EDS) results obtained from HRSEM at the (**a**) bare SPCE and (**b**) NGr-DMG-SPCE.

The surface morphology of the modified electrodes was studied using HRSEM analysis in conjunction with energy dispersive spectroscopy (EDS) for confirming the presence of graphene and dimethylglyoxime at the electrode surface. High resolution scanning electron microscope images of bare screen printed carbon electrodes (SPCE) and nafion-graphene dimethylglyoxime modified screen printed electrodes (NGr-DMG-SPCE) are shown in Figure 4 at 1000× and 20,000× magnification. Samples for HRSEM were prepared by drop coating 4 µL of NGr-DMG nanocomposite directly onto the cleaned SPCE and allowing it to dry at room temperature. Improved imaging is achieved by sputter coating of a Au-Pd film (≤5 nm) onto the dried SPCE surface to enhance surface conductivity. The bare SPCE (Figure 4a,c) shows a rough, porous surface, typically associated with most carbon structures as a result of the printed carbon ink. No other significant features are observed. In contrast, Figure 4b,d), show a smooth, uniform, solid surface HRSEM images for the NGr-DMG-SPCE surface, at both low and high magnifications. This may be attributed to the presence of a film at the electrode surface, due to the incorporation of Nafion. Furthermore, flake-like features, associated with the presence of graphene on the electrode surface can be seen. The changes in surface morphology of the electrode surface confirm the presence of the nafion-graphene dimethylglyoxime film on the SPCE. Elemental analysis of the bare and modified SPCE surface was performed by energy dispersive spectroscopy (EDS). Figure 5, represents the recorded EDS data obtained from HRSEM analysis. The bare SPCE (Figure 5a) shows the presence of C, O and Cl at 0.3, 0.5 and 2.6 keV, associated with the conductive carbon ink used to create the carbon electrode. Gold sputter coating was employed for the HRSEM analysis and is present at the electrode surface. Analysis of the modified NGr-DMG-GCE (Figure 5b) shows the inclusion of N and F at 0.4 and 0.6 keV and an increase in the C and O elemental intensities when compared to the bare SPCE. The presence of N and a significant increase in C and O intensities in the EDS spectrum confirm the presence of both Nafion and DMG at the NGr-DMG-SPCE surface, while fluorine is associated with the Nafion binder.

### 3.5. Electrochemical Characterization of the Nafion-Graphene Dimethylglyoxime Modified Glassy Carbon Electrode (NGr-DMG-GCE)

**Figure 6:**
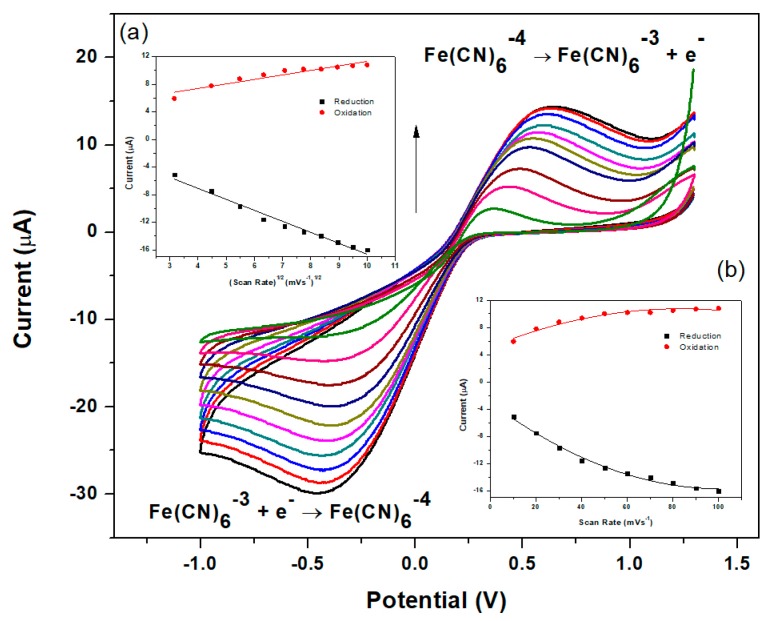
Electrochemical characterization of N-DMG-GCE in 5 mM Fe(CN)_6_^3−/4−^ at scan rate of 10–100 mV s^−1^ in supporting electrolyte (1 M KCl). Insets: plots of peak currents vs. (**a**) scan rate1/2 and (**b**) scan rate of the main oxidation and reduction waves.

**Figure 7:**
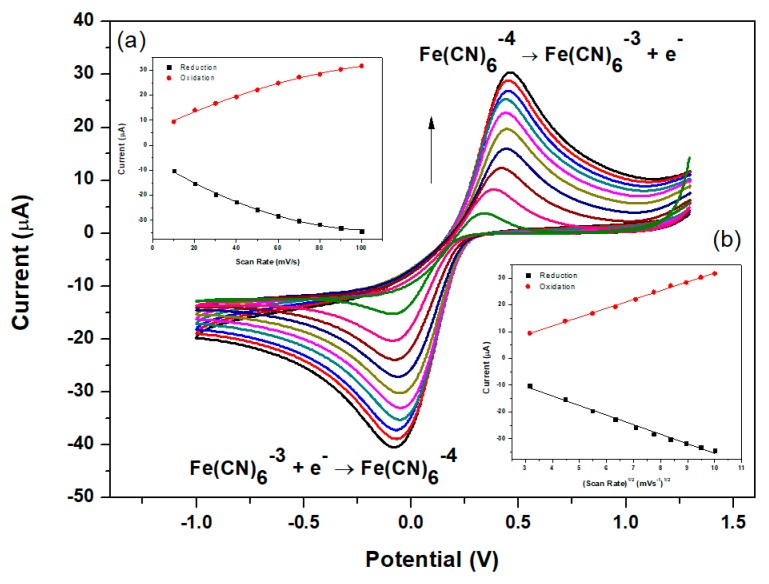
Electrochemical characterization of NGr-DMG-GCE in 5 mM Fe(CN)_6_^3−/4−^ at scan rate of 10–100 mV s^−1^ in supporting electrolyte (1 M KCl). Insets: plots of peak currents vs. (**a**) scan rate and (**b**) scan rate1/2 of the main oxidation and reduction waves.

The electrochemical properties related to the active surface area of the modified glassy carbon electrodes were characterized by cyclic voltammetry (CV). Since the NGr-DMG-GCE demonstrates no significant redox peaks in the potential window of interest, a ferro/ferricyanide (Fe(CN)_6_^3−/4−^) redox couple, giving rise to a reversible one electron system was used to study the electrochemical electrode properties. Cyclic voltammograms obtained for the N-DMG-GCE and NGr-DMG-GCE at various scan rates were recorded in the presence of a 5 mM K_3_Fe(CN)_6_ solution and illustrated in Figure 5 and Figure 6, respectively. The CV for the NGr-DMG-GCE demonstrates single oxidation and reduction peaks in the potential range between −1.0 and +1.25 V. An increase in the anodic and cathodic peak currents was observed with a slight shift to more positive and negative potentials with increasing scan rates between 10 and 100 mV s^−1^. The variation of peak current versus (a) scan rate and (b) scan rate^1/2^ is reported in Figure 6 and Figure 7, insets respectively and allows for discrimination between adsorption and diffusion controlled processes. A linear variation of peak currents with the square root of scan rates is observed at both modified electrodes and suggests a diffusion controlled process of Fe(CN)_6_^3−/4−^ ions at both the N-DMG-GCE and NGr-DMG-GCE surfaces [53].

The diffusion coefficients through the N-DMG-GCE and NGr-DMG-GCE’s were determined by studying the cyclic voltammetric response of the reversible ferro/ferricyanide (Fe(CN)_6_^3−/4−^) system using the Randles-Sevcik equation:

(3) Ipf=(2.69×105)n32AD12C*υ12

where *I_pf_* is peak current (*A*), n is the number of electrons transferred, *A* is the active area of the working electrode (cm^2^), *D* is the diffusion coefficient (cm^2^ s^−1^), C* is the bulk concentration of the electroactive species (mol cm^−3^) and v is the potential scan rate (V s^−1^). The diffusion coefficients of the Fe(CN)_6_^3−/4−^ ions at the N-DMG-GCE and NGr-DMG-GCE were calculated as 4.88 × 10^−8^ and 1.13 × 10^−6^ cm^2^s^−1^ respectively. A 23 times increase in diffusion rate is observed for the NGr-DMG-GCE compared with the N-DMG-GCE. This significant increase in rate of diffusion occurs as a result of a significant increase in active surface area attributed to graphene loading [54].

### 3.6. Electrochemical Behaviour of the NGr-DMG-GCE

**Figure 8:**
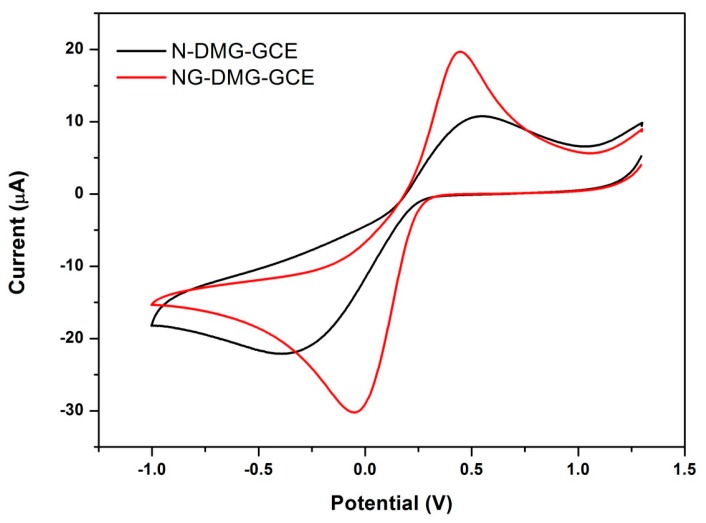
Cyclic voltammograms of 5 mM Fe(CN)_6_^3−/4−^ at N-DMG-GCE (black) and NGr-DMG-GCE (red) in supporting electrolyte of 1 M KCl at scan rate 50 mV s^−1^.

Cyclic voltammograms recorded in 5 mM Fe(CN)_6_^3−/4−^ solution with 1 M KCl as supporting electrolyte and at a scan rate of 50 mV s^−1^ for the N-DMG-GC and NGr-DMG-GC electrodes are shown in Figure 8. A pair of distinct, well-defined redox couple peaks for Fe(CN)_6_^3−/4−^ are observed between −1.0 V and +1.25 V in a 1 M KCl solution. Two broad peaks are observed at the N-DMG-GCE, with a large formal potential (ΔE_p_) of 0.45 V. The large ΔE_p_ is as a result of diffusion controlled processes dominating over the electrochemical reduction of the Fe(CN)_6_^3−/4−^ couple. This is expected owing to the organic nature of the DMG ligand which hampers electron transfer [49]. However, a decrease to 0.25 V in the formal potential for the NGr-DMG-GCE was observed. The NGr-DMG-GCE showed a distinct increase in peak currents of the redox peaks as well as, a narrowing in peak separation when compared with the N-DMG-GCE, as observed by the lower E_p_ value, namely from 1.07 to 0.53 V. This decrease in peak separation is attributed to the improved electrical properties due to electron transfer processes intrinsic with graphene. In addition the capacitive background current also increased showing improvement of the non-electroactive surface area due to graphene modification [54]. This result favors the use of the NGr-DMG complex as a modifier for the electrochemical sensing application.

### 3.7. Electrochemical Impedance Spectroscopy (EIS) Analysis of the NGr-DMG-GCE

**Figure 9:**
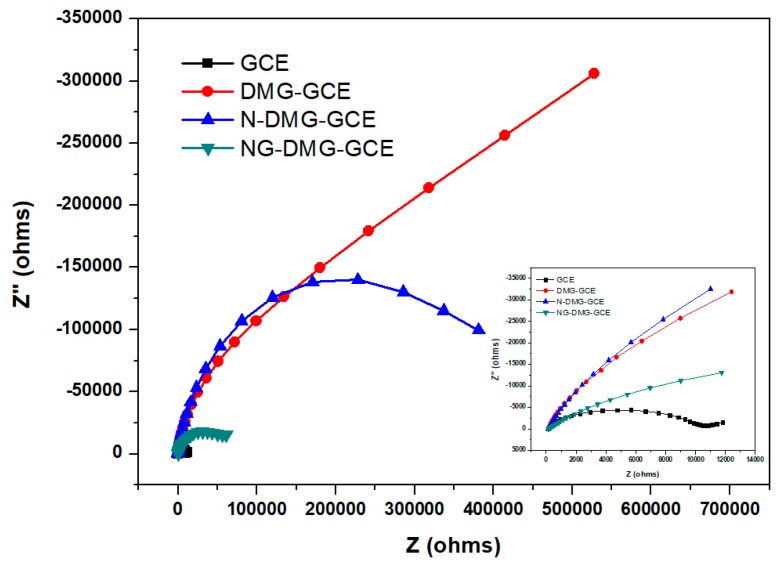
Nyquist plots at GCE (black); DMG-GCE (red); N-DMG-GCE (blue) and NGr-DMG-GCE (green) in 5 mM Fe(CN)_6_^3−/4−^ containing 1 M KCl. Inset is the magnified Nyquist plot between 0 and 12 kΩ. The frequency range is between 0.1 and 100,000 Hz.

Further investigation of the electron transfer properties at the bare and modified electrodes were performed using electrochemical AC impedance experiments. Here, the changes in the impedance and interface properties during the electrode surface modification procedure were measured and are shown in Figure 9. All electrochemical impedance spectroscopy (EIS) experiments were carried out in the presence of a 5 mM Fe(CN)_6_^3−/4−^ redox probe over the 0.1–100,000 Hz range. Typically, impedance spectra consist of semi-circular and linear regions which represent electron transfer and diffusion processes, respectively. Electron transfer processes occur at higher frequencies, whilst the diffusion processes occur at lower frequencies.

Nyquist plots, Figure 9, were obtained by plotting imaginary impedance, Z’’ versus real impedance, Z”, for the bare GCE, DMG-GCE, N-DMG-GCE and NGr-DMG-GCE and hence, considerable changes in the diameter of the semicircle were observed upon electrode modification. Furthermore, a large increase in charge transfer resistance (R_ct_), was observed upon the inclusion of the non-conductive dimethylglyoxime chelating agent onto the GCE whilst, the infinitely large semicircle suggests very slow electron transfer processes and an increase in the total impedance. The modification of GCEs with both Nafion and a Nafion-graphene nanocomposite leads to an effective lowering of the recorded R_ct_ values. The lower R_ct_ values of the NGr-DMG-GCE demonstrates a higher electrochemical activity and enhanced electron transfer kinetics across the electrode-solution interface when compared with the DMG and Nafion modified electrodes owing to the high conductivity and improved surface area to volume ratio of graphene. This confirms the findings in Figure 8 above. In general, the linear region in the Nyquist plot is linked to reactions where mass-transfer (Warburg Impedance) controls the reaction rate and electron transfer is slow. The absence of a linear diffusion region in the DMG-GCE and N-DMG-GCE may be attributed to the dominance of slow electron transfer over the diffusion controlled processes.

### 3.8. Further Electrochemical Characterization of the NGr-DMG-GCE

**Figure 10:**
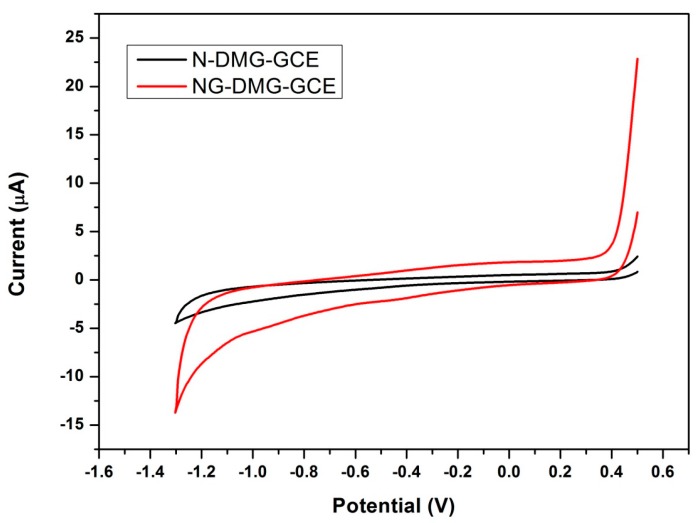
Cyclic voltammograms of N-DMG-GCE (black) and NGr-DMG-GCE (red). Supporting electrolyte 0.1 M NH_3_/NH_4_Cl Buffer (pH 9.3), scan rate (10 mV s^−1^), deposition time (120 s), frequency (20 Hz), amplitude (0.02 V) and voltage step (0.005 V).

The electrochemical properties of the N-DMG-GCE and NGr-DMG-GCE were further interrogated using cyclic voltammetry the resulting voltammograms are shown in Figure 10. Both modified electrodes show no significant redox peaks are present in the potential window under investigation. An increase in the background current is evident in the voltammogram of the NGr-DMG-GCE when compared to the N-DMG-GCE. This phenomenon indicates the increase of active surface area and conductivity of the modified electrode as a result of the graphene coating.

### 3.9. Effect of the NGr-DMG-GCE on the Stripping Response of Ni^2+^

**Figure 11:**
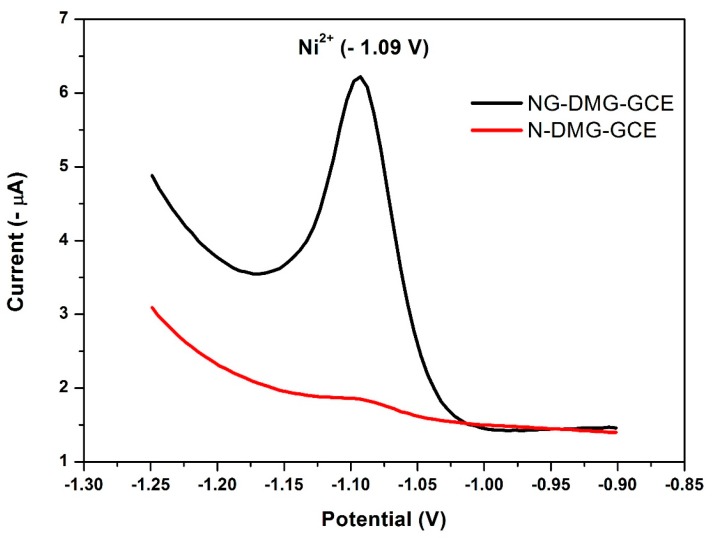
SW-AdCSV of 20 μg L^−1^ Ni^2+^ at N-DMG-GCE (red) and NGr-DMG-GCE (black) with a characteristic reduction stripping potential of −1.09 V. Supporting electrolyte (0.1 M NH_3_/NH_4_Cl Buffer (pH 9.3)), deposition potential (−0.7 V), deposition time (120 s), frequency (20 Hz), amplitude (0.02 V) and voltage step (0.005 V).

Figure 11 represents the influence of graphene in the NGr-DMG nanocomposite on the stripping response of Ni^2+^. The N-DMG-GCE shows a broad, small peak at −1.08 V in 0.1 M NH_3_/NH_4_Cl Buffer (pH 9.3). In contrast, the NGr-DMG-GCE shows a sharp, well-resolved stripping peak at −1.09 V for Ni^2+^ reduction from the Ni(DMGH)_2_ adsorption complex as demonstrated in Equation (2).

A considerable increase (nine times) in stripping peak current was observed for the NGr-DMG-GCE in comparison to the N-DMG-GCE with no shift in redox half-wave potential. This increase may be attributed to the increase in active surface area and quantum confinement of the electrode due to the incorporation of conductive, graphene sheets.

### 3.10. Influence of Dimethylglyoxime Concentration

**Figure 12:**
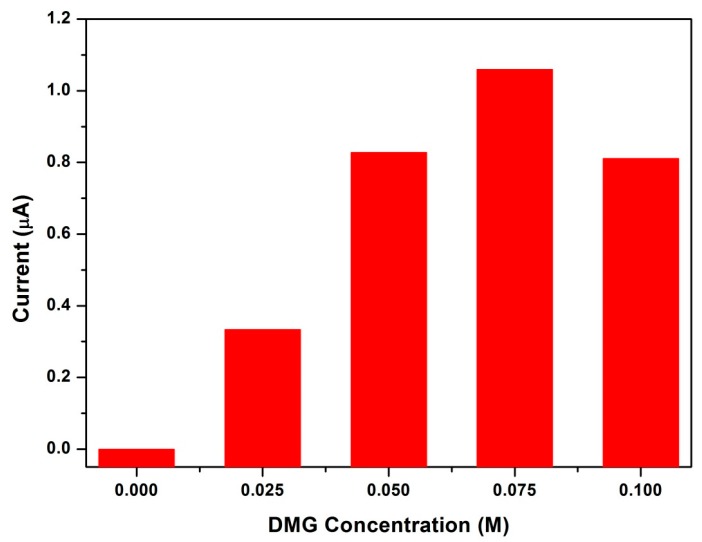
Influence of dimethylglyoxime concentration (0–0.1 M) on the stripping peak currents of Ni^2+^ at the NGr-DMG-GCEs in 0.1 M NH_3_/NH_4_Cl buffer (pH 9.3).

The reduction of Ni^2+^ from the electrochemical probe is dependent on two important criteria; firstly, the adsorption of the metal ions onto the electrode surface (Equation (1)) and secondly the speed and ease of electron transfer through the NGr-DMG coating. The influence of dimethylglyoxime concentration on the stripping peak current of Ni^2+^ within the NGr-DMG nanocomposite was investigated and reported in Figure 12. The concentration of dimethylglyoxime was varied between 0.0 and 0.1 M. No stripping peak current for Ni^2+^ was observed in the absence of DMG, however a constant increase in stripping peak response is then observed with increasing dimethylglyoxime concentration in the coating solution between 0.0 and 0.075 M. Saturation of the electrode surface at DMG concentrations greater than 0.075 M is evident followed by a sharp decrease in the resultant stripping peak current. Here, blocking of electron transfer reactions through the DMG nanocomposite film occurs due to its considerably high DMG content. A concentration of 0.075 M dimethylglyoxime in the NGr-DMG nanocomposite was optimum and thus utilized for all further experiments.

### 3.11. Optimization of Instrumental Parameters

**Figure 13:**
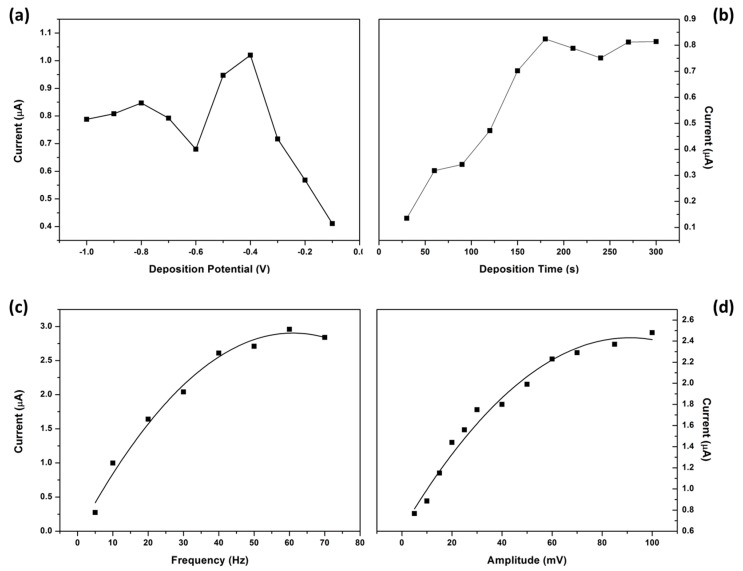
Effect of instrumental parameters; (**a**) deposition potential; (**b**) deposition time; (**c**) frequency and (**d**) amplitude on the stripping peak current of 10 µg L^−1^ Ni^2+^ at NGr-DMG-GCE. Supporting electrolyte (0.1 M NH_3_/NH_4_Cl Buffer (pH 9.3)).

The square wave instrumental parameters affecting the analytical response of the NGr-DMG-GCE; namely deposition potential, deposition time, frequency and amplitude were optimized and illustrated in Figure 13.

The influence of deposition potential on the stripping response of Ni^2+^ at the NGr-DMG-GCE was interrogated in the potential range between 0.0 and −1.0 V, more positive than the half-wave potential of Ni^2+^, Figure 13a. The deposition potential is responsible for aiding in the accumulation of the analyte at the electrode surface. The stripping response increased to a maximum at −0.4 V, due to the formation of the Ni(DMGH)_2_ adsorption complex. A gradual decrease is observed at potentials more negative than −0.4 V as the Ni^2+^ half-wave potential is approached. A deposition potential of −0.7 V was selected for all further experiments.

The effect of deposition/accumulation time on the Ni^2+^ stripping response was studied between 30 and 300 s. Figure 13b, shows a rapid increase in the Ni^2+^ peak current with increasing accumulation time between 0 and 180 s confirming the increase in adsorption of the Ni^2+^ on the NGr-DMG-GCE surface. Saturation of the NGr-DMG-GCE takes place at accumulation times greater than 180 s and results in a decrease in stripping peak current. A deposition time of 120 s was selected for all analysis.

Figure 13c shows the dependence of peak currents on the square wave frequency over the 5 to 70 Hz range. The peak current increases with increasing frequency to 60 Hz, before gradually decreasing. The increase in peak current is as a result of the increase in scan rate with increasing frequency. A frequency of 50 Hz was selected for further study. The influence of amplitude on the stripping peak current of Ni^2+^ was studied between 5 and 100 mV and shown in Figure 13d. The stripping peak current increases with increasing amplitude hence an amplitude of 40 mV was selected for all further experiments.

### 3.12. Influence of Electrolyte pH

**Figure 14:**
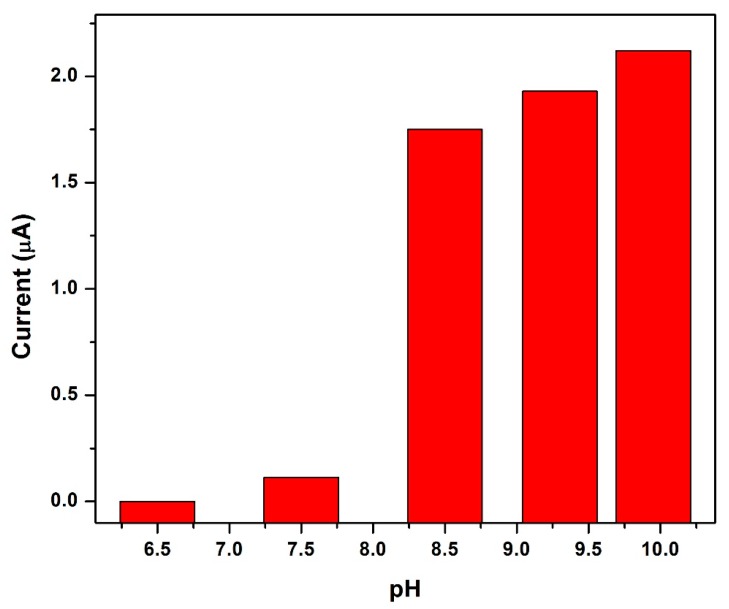
Dependence of electrolyte pH on the stripping peak currents of Ni^2+^ at the NGr-DMG-GCEs in 0.1 M NH_3_/NH_4_Cl buffer as electrolyte solution.

The formation of the Ni(DMGH)_2_ complex and its adsorption, is the determining step in Ni^2+^ detection by AdSV and is controlled by the electrolyte solution and its pH The influence of the electrolyte solution pH on the stripping peak current of Ni^2+^ was investigated between pH 6–10 and is presented in Figure 14. No significant stripping peak for Ni^2+^ was found at the NGr-DMG-GCE for pH values below 8 indicating very little complex formation. A drastic increase in peak currents between pH values of 8.5 and 10 can be seen confirming complex formation. A minimum pH value of 8.5 is therefore required to facilitate the formation of the Ni(DMGH)_2_ complexes at the NGr-DMG-GCE surface. A pH of 9.2 was selected for all further experiments.

### 3.13. Influence of Oxygen Removal

**Figure 15:**
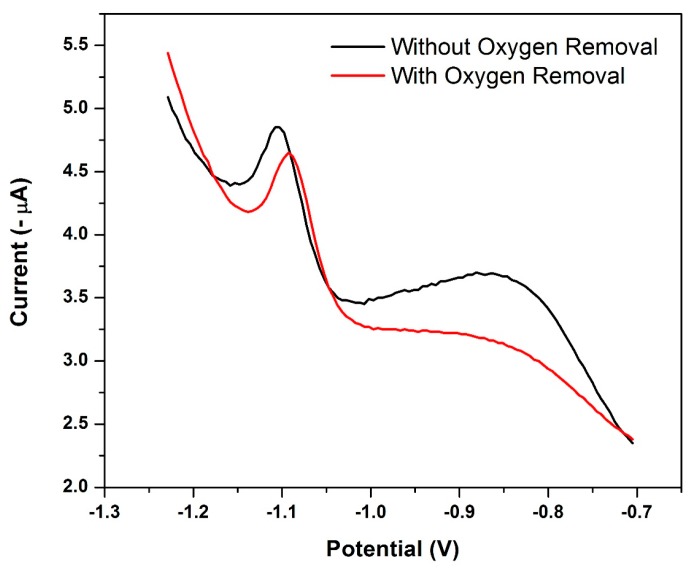
Square-Wave Voltammogram of 4 µg L^−1^ Ni^2+^ at a NGr-DMG-GCE before deoxygenation (black) and after deoxygenation (red). Supporting electrolyte (0.1 M NH_3_/NH_4_Cl Buffer (pH 9.3)), deposition potential (−0.7 V), deposition time (120 s), frequency (20 Hz), amplitude (0.02 V) and voltage step (0.005 V).

Rapid analysis times of the square-wave waveform limits the influence of the irreversible oxygen reduction current. The influence of deoxygenation of the sample on the cathodic stripping peak current of Ni^2+^ reduction at the NGr-DMG-GCE is shown in Figure 15. Deoxygenation was achieved by bubbling N_2_ gas through the sample for 5 min. A large broad peak between −0.7 and −1.0 V was observed at the NGr-DMG-GCE before oxygen removal and can be attributed to the reduction wave of dissolved oxygen. An insignificant increase in the Ni^2+^ stripping peak response after oxygen removal indicates no discernable interference of oxygen on the measurement, owing to the cation reduction being close to the oxygen reduction wave. Similar results are obtained in the work by Zen et al. [23,24].

A slight shift in peak potential of the Ni^2+^ reduction peak from −1.105 V to more positive oxidation potentials of −1.092 V is observed after oxygen removal due to improved electron transfer processes. All samples were therefore degassed under N_2_ for 5 min prior to use in order to minimize any further influence from oxygen on the stripping peak potentials.

### 3.14. Electrode Reproducibility and Interference Studies

**Figure 16:**
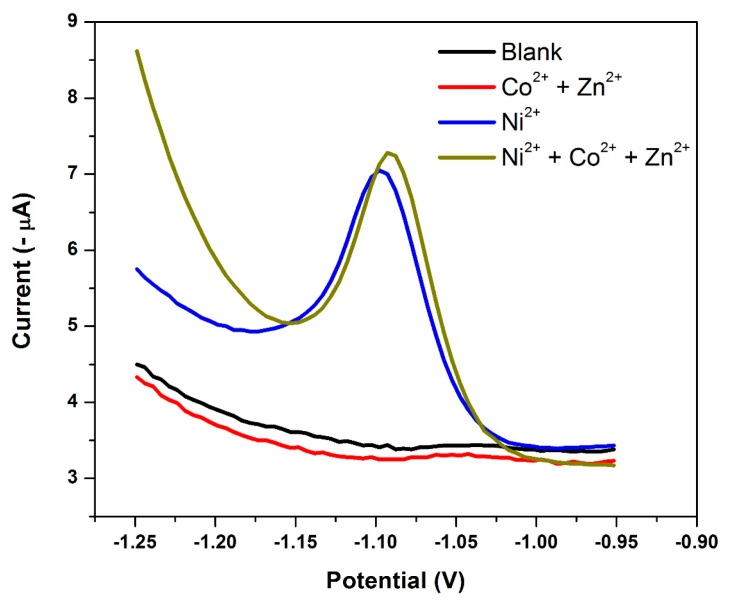
Square-wave voltammograms of 0 µg L^−1^ metal cations (black); 200 µg L^−1^ Co^2+^ and Zn^2+^ (red); 20 µg L^−1^ Ni^2+^ (blue) and 20 µg L^−1^ Ni^2+^ in the presence of 200 µg L^−1^ Co^2+^ and Zn^2+^ (green) at a NGr-DMG-GCE. Supporting electrolyte (0.1 M NH_3_/NH_4_Cl buffer (pH 9.3)), deposition potential (−0.7 V), deposition time (120 s), frequency (20 Hz), amplitude (0.02 V) and voltage step (0.005 V).

The selectivity of the NGr-DMG-GCE sensor was investigated by studying the stripping voltammetric determination of 20 µg L^−1^ Ni^2+^, under optimized conditions in the presence of Co^2+^ and Zn^2+^ cations. Co^2+^ and Zn^2+^, were selected as possible interferents due to their peak potentials being close to Ni^2+^ and within the potential range under investigation. Furthermore, Co^2+^ readily forms complexes with dimethylglyoxime under similar conditions. All other metallic cations were not included for the interference studies. Figure 16, depicts the voltammograms recorded at the NGr-DMG-GCEs for (a) 0 µg L^−1^ Ni^2+^, Co^2+^ and Zn^2+^; (b) 200 µg L^−1^ Co^2+^ and Zn^2+^; (c) 20 µg L^−1^ Ni^2+^ and (d) 20 µg L^−1^ Ni^2+^ in the presence of 200 µg L^−1^ Co^2+^ and Zn^2+^. No stripping peaks were found in the absence of metal cations between −0.7 and −1.3 V, as expected. Similarly, Co^2+^ and Zn^2+^ were also found to exhibit no reduction stripping peaks at the NGr-DMG-GCE between 0 and 200 µg L^−1^. This result differs significantly from previous research on metal-based sensors where Co^2+^ has been detected in the presence of DMG and a metallic film, and could be attributed to the selective non-binding of Co^2+^ and Zn^2+^ to the modified electrode surface. This is a significant finding since it improves the electrode selectivity towards Ni^2+^ detection in the presence of Co^2+^ and Zn^2+^. The determination of Ni^2+^ in 0.1 M NH_3_/NH_4_Cl Buffer (pH 9.3) under optimum conditions show a reproducible and, well-defined stripping peak for Ni^2+^ reduction from the Ni(DMGH)_2_ complex. Hence, the Ni(DMGH)_2_ detection is therefore independent of mercury or metallic film and relies solely on the metal-chelate complex formation. The results are further summarized in Table 1.

Cation concentrations which resulted in variations in stripping peak currents greater than 5% for three successive replicas (*n* = 3) were considered as interferents. The NGr-DMG-GCE is shown to have sensitivity 10 times greater than that of the N-DMG-GCE. The peak current for Ni^2+^ reduction remains constant for the detection of 20 µg L^−1^, with a relative standard deviation (RSD %) of 5.46% thus confirming a highly reproducible system. The stripping peak currents of Ni^2+^ in the presence of excess Co^2+^ and Zn^2+^ up to 10 times the Ni^2+^ concentration was investigated and a RSD (%) of 3.94% was obtained. The results indicate that the NGr-DMG-GCE sensor is highly reproducible for Ni^2+^ determination and shows good stability. It further indicates that no significant interferences from Co^2+^ and Zn^2+^ on the Ni^2+^ stripping peak up to 10 times the analyte concentration was observed. The NGr-DMG-GCE sensor can thus be applied for Ni^2+^ detection in water samples in which Co^2+^ and Zn^2+^ ions are also present.

### 3.15. Analytical Performance of the NGr-DMG-GCE

**Figure 17:**
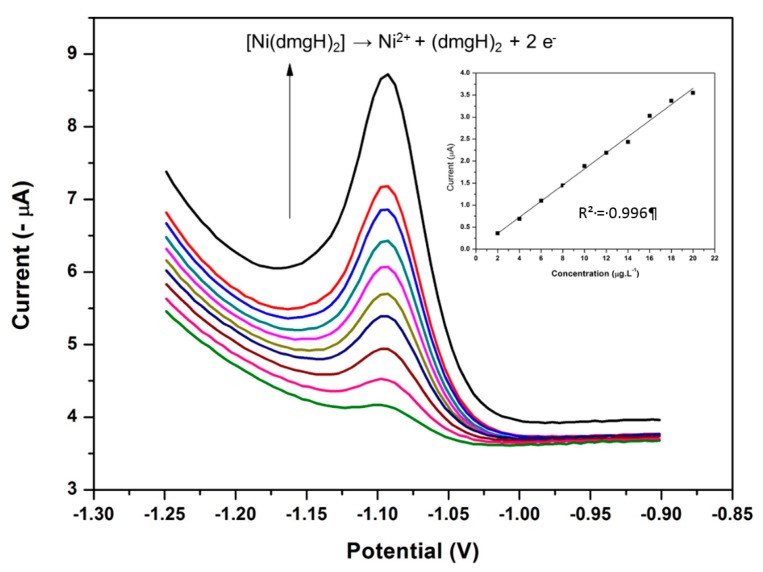
SWAdSV and corresponding calibration plot of the individual analysis of Ni^2+^ in the presence of Co^2+^ and Zn^2+^ obtained at a NGr-DMG-GCE over 2–20 µg L^−1^. Supporting electrolyte (0.1 M NH_3_/NH_4_Cl Buffer (pH 9.3)), deposition potential (−0.7 V), deposition time (120 s), frequency (20 Hz), amplitude (0.02 V) and voltage step (0.005 V).

The analytical performance of the NGr-DMG-GCE sensor was evaluated for Ni^2+^ detection in test samples under optimum conditions. The recorded square-wave stripping voltammograms from the sensor and the corresponding calibration plots for the analysis of Ni^2+^ in 0.1 M NH_3_/NH_4_Cl buffer solution (pH 9.3) containing Co^2+^ and Zn^2+^ at a NGr-DMG-GCE for 120 s are shown in Figure 17. The sensor showed a linear increase in the Ni^2+^ reduction peak current with increasing Ni^2+^ concentration over a 2–20 µg L^−1^ concentration range. The limit of detection (LOD) of the Ni^2+^ sensor in the presence of Co^2+^ and Zn^2+^ were determined according to Equation (4):

(4) $$\mathrm{LOD}=\frac{3\sigma }{\mathrm{slope}}$$

where, LOD is the limit of detection; 3σ is three times the standard deviation of the blanks; slope is the gradient (slope) of the calibration curve.

The standard deviation of the blanks was determined from 10 replications of the NGr-DMG-GCE in 0.1 M NH_3_/NH_4_Cl Buffer (pH 9.3) in the presence of excess Co^2+^ and Zn^2+^. A summary of previously reported electrochemical sensors for the detection of Ni^2+^ in water at: (a) chemically modified DMG electrodes and (b) in-situ deposited DMG electrodes as well as a summary of detection limits from this work are reported in Table 2 below. It was observed that the in-situ deposition of metal-DMG complexes onto metallic films provides more sensitive electrochemical sensors as seen by its significantly lower detection limits and faster analysis times compared to that of chemically modified DMG electrodes. This is to be expected due to the presence of excess chelating agent for complexation in solution as well as the presence of a metal film for adsorption of metal-chelate complexes. The use of chemically modified DMG electrodes with mercury films gives better detection limits over its metal-free counterparts. The use of graphene in our study has shown to significantly lower the detection limits by improving electrode sensitivity in comparison to previously used chemically modified DMG metal-free sensors and, has for the first time shown that Ni^2+^ can be detected in the presence of Co^2+^ and Zn^2+^. A limit of detection of 1.5 μg L^−1^ was found for Ni^2+^ detection at 120 s analysis time. Lower LODs can be found at longer accumulation times.

### 3.16. Application of the NGr-DMG-GCE to Real Water Samples

**Figure 18:**
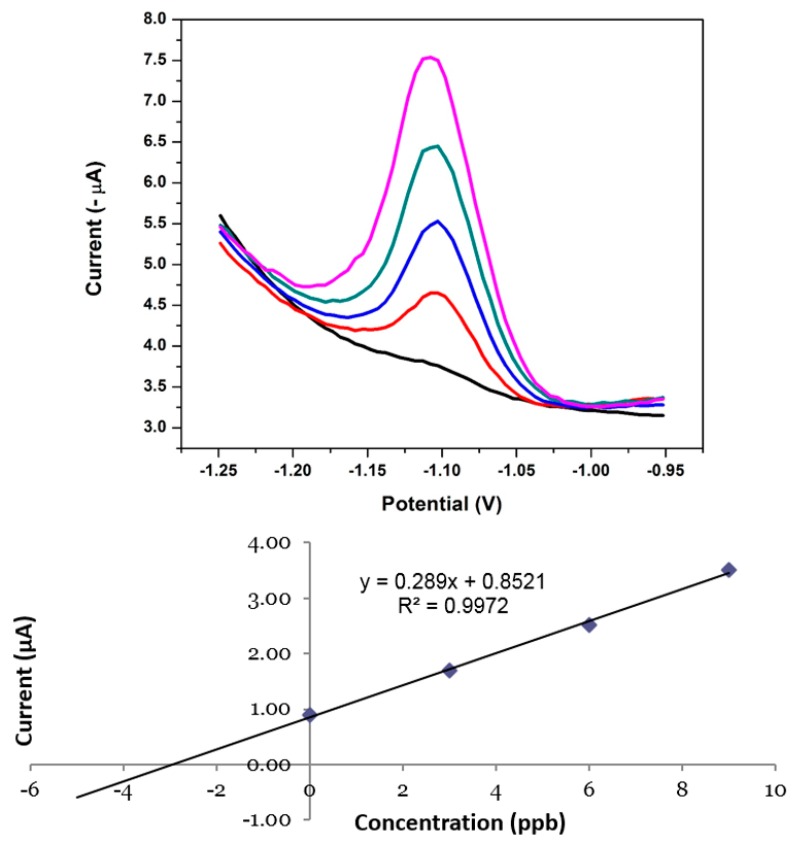
SWAdSV and corresponding standard addition calibration plot of the individual analysis of Ni^2+^ in the presence of Co^2+^ and Zn^2+^ obtained at a NGr-DMG-GCE in real tap water samples. Supporting electrolyte (0.1 M NH_3_/NH_4_Cl buffer (pH 9.3)), deposition potential (−0.7 V), deposition time (120 s), frequency (20 Hz), amplitude (0.02 V) and voltage step (0.005 V).

In order to further investigate the analytical performance and accuracy of the NGr-DMG-GCE sensor towards the detection of Ni^2+^ in the presence of excess Co^2+^ and Zn^2+^, recovery studies were performed in both test and real water samples using the standard addition method. Tap water samples were collected in our laboratory and investigated for the detection of Ni^2+^. The recorded voltammograms and associated standard addition calibration plot are illustrated in Figure 18 and a summary of the recovery percentages obtained from the analysis of tap water and test solutions is shown in Table 3. No Ni^2+^ was detected in either test or real water samples at 120 s preconcentration time. This distinct absence of reduction peaks suggests that the concentration of metal ions was below the sensor’s limit of detection. However, when known concentrations (3 µg L^−1^) of Ni^2+^ was spiked into water samples in the presence of Co^2+^ and Zn^2+^ and detected by performing consecutive additions of known concentration to the ‘unknown’ sample (standard addition method) from 3–12 µg L^−1^. Ni^2+^ yielded accurate recovery percentages in both test and real water samples with errors within the acceptable sensor limits. The results verify the use of the sensor in water samples with low concentrations of Ni^2+^.

## 4. Conclusions

For the first time a Nafion-graphene dimethylglyoxime modified glassy carbon electrode was interrogated for the trace determination of Ni^2+^ in tap water samples. The fabrication of the DMG modified probe showed a green approach towards Ni^2+^ detection since the need for toxic metal films commonly used in metal analysis was eliminated. Graphene synthesized by a chemical synthesis method further showed enhanced electrode sensitivity towards Ni^2+^ detection owing to improved electron transfer kinetics and the increase in active surface area of the electrode surface. The NGr-DMG-GCE probe further showed superior sensitivity and selectivity towards detection of Ni^2+^ in the presence of common interferents such as Co^2+^ and Zn^2+^ ions. Detection limits in the low parts per billion range (1.5 µg L^−1^), that are well below the USEPA and WHO maximum contamination limits was achieved at short pre-concentration times (120 s). The NGr-DMG-GCE was successfully applied to the detection of Ni^2+^ in tap water samples with recoveries within 5–10% error.

## Figures and Tables

**Table 1:** Reproducibility and interference studies of the N-DMG-GCE and NGr-DMG-GCE (*n* = 3).

Substrate	Analyte	Concentration (µg L^−1^)	Peak Current (A)	Std. Dev. (A)	% RSD
N-DMG-GCE	Co^2+^ and Zn^2+^	200	N/D	N/D	N/D
	Ni^2+^	20	1.12 × 10^−7^	1.12 × 10^−8^	3.72
	Ni^2+^ in the presence of Co^2+^ and Zn^2+^	20	1.09 × 10^−7^	1.63 × 10^−8^	4.01
NGr-DMG-GCE	Co^2+^ and Zn^2+^	200	N/D	N/D	N/D
	Ni^2+^	20	1.94 × 10^−6^	1.06 × 10^−7^	5.46
	Ni^2+^ in the presence of Co^2+^ and Zn^2+^	20	1.87 × 10^−6^	1.34 × 10^−7^	3.94

**Table 2:** A summary of selected previously reported (a) in-situ DMG and (b) chemically modified DMG sensors for adsorptive stripping voltammetric detection of Ni^2+^.

	Metal Ions	Substrate	Technique	Accumulation Time (min)	Dynamic Linear Range (µg L^−1^)	Detection Limit (µg L^−1^)	Reference
In-situ DMG Electrodes	Ni^2+^	mpBiF-SPCE	AdCSV	180	1–10	0.027	[28]
	Co^2+^				1–10	0.094	
	Ni^2+^	RBiABE	DPAdSV	30	0.6–41	0.18	[14]
	Co^2+^				0.06–4.1	0.018	
	Ni^2+^	PbF-SPE	SWV	60	0.6–2.9	0.2	[15]
	Co^2+^				0.6–5.9	0.3	
	Ni^2+^	SBVE	SW-AdCSV	30	0–10	0.6	[55]
Chemically Modified DMG Electrodes	Ni^2+^	NC-DMG-MFE	SWASV	300	0.1–100	0.1	[22]
	Cu^2+^				1.0–80	1.0	
	Ni^2+^	MCPE	AAdSV	720	6–600	0.006	[18]
	Ni^2+^	DMG-CPE	DPV	1500	80–600	27	[20]
	Ni^2+^	DMG-N/SPE	DPV	120	60–500	30	[16]
	Ni^2+^	PVC-PA-DMG-GCE	SWAdCSV	240	18–180	18	[8]
	Ni^2+^ with Co^2+^ and Zn^2+^	NGr-DMG-GCE	SWAdSV	120	2–20	1.5	This Work

**Table 3:** Recovery studies of the NGr-DMG-GCE in test and tap water samples.

Ni^2+^ Sample	Original (µg L^−1^)	Added (µg L^−1^)	Found (µg L^−1^)	RSD (%)	Recovery (%)
Test Sample	N/D	3.00	3.16	8.21	105
Real Water Sample	N/D	3.00	3.35	7.46	111
